# Molecular Characterization of Mg-Chelatase CHLI Subunit in Pea (*Pisum sativum* L.)

**DOI:** 10.3389/fpls.2022.821683

**Published:** 2022-01-25

**Authors:** Cai-jun Wu, Jie Wang, Jun Zhu, Jing Ren, You-xin Yang, Tao Luo, Lu-xi Xu, Qing-hong Zhou, Xu-feng Xiao, Yu-xin Zhou, Sha Luo

**Affiliations:** ^1^Department of Horticulture, College of Agronomy, Jiangxi Agricultural University, Nanchang, China; ^2^Institute of Life Science and School of Life Sciences, Nanchang University, Nanchang, China

**Keywords:** chlorophyll synthesis, Mg-chelatase CHLI subunit, photosynthesis, *Pisum sativum*, protein-protein interaction, virus-induced gene silencing

## Abstract

As a rate-limiting enzyme for chlorophyll biosynthesis, Mg-chelatase is a promising target for improving photosynthetic efficiency. It consists of CHLH, CHLD, and CHLI subunits. In pea (*Pisum sativum* L.), two putative *CHLI* genes (*PsCHLI1* and *PsCHLI2*) were revealed recently by the whole genome sequencing, but their molecular features are not fully characterized. In this study, *PsCHLI1* and *PsCHLI2* cDNAs were identified by PCR-based cloning and sequencing. Phylogenetic analysis showed that PsCHLIs were derived from an ancient duplication in legumes. Both *PsCHLIs* were more highly expressed in leaves than in other organs and downregulated by abscisic acid and heat treatments, while *PsCHLI1* was more highly expressed than *PsCHLI2*. *PsCHLI1* and *PsCHLI2* encode 422- and 417-amino acid proteins, respectively, which shared 82% amino acid identity and were located in chloroplasts. Plants with a silenced *PsCHLI1* closely resembled *PsCHLI1* and *PsCHLI2* double-silenced plants, as both exhibited yellow leaves with barely detectable Mg-chelatase activity and chlorophyll content. Furthermore, plants with a silenced *PsCHLI2* showed no obvious phenotype. In addition, the N-terminal fragment of PsCHLI1 (PsCHLI1N, Val63-Cys191) and the middle fragment of PsCHLI1 (PsCHLI1M, Gly192-Ser336) mediated the formation of homodimers and the interaction with CHLD, respectively, while active PsCHLI1 was only achieved by combining PsCHLI1N, PsCHLI1M, and the C-terminal fragment of PsCHLI1 (Ser337-Ser422). Taken together, PsCHLI1 is the key CHLI subunit, and its peptide fragments are essential for maintaining Mg-chelatase activity, which can be used to improve photosynthetic efficiency by manipulating Mg-chelatase in pea.

## Introduction

Pea (*Pisum sativum* L.) is the second most important legume crop in the world and produces high protein feed for animal and human nutrition (Kreplak et al., 2019). It is also a valuable source of mineral nutrients, complex starch, several vitamins, antioxidants, and fiber, which are fairly low in calories and demonstrate health benefits (Guillon and Champ, 2003; Dahl et al., 2012; Tayade, 2019). The growth of the demand for peas accompanies with the increasing world population, mainly in developing regions. To meet the growing need for peas, yield and quality must be improved at a higher rate than before by agricultural approaches.

There is a direct cause-effect relationship between crop production and photosynthesis (Moss and Musgrave, 1971), and many efforts have been made to achieve greater yield by improving photosynthetic efficiency (Zhu et al., 2010; Long et al., 2015). Various factors can affect photosynthetic efficiency, and chlorophyll content is essential (Croce and van Amerongen, 2014). The chlorophyll biosynthesis pathway is the Mg^2+^ branch of the tetrapyrrole biosynthetic pathway, which is catalyzed by a heterotrimeric enzyme, Mg-chelatase, composed of CHLI (36–46 kDa), CHLD (60–87 kDa), and CHLH (120–155 kDa) subunits (Masuda, 2008). The catalytic properties of plant Mg-chelatase were first revealed in pea (Walker and Weinstein, 1994; Guo et al., 1998). The enzymatic reaction includes at least two steps, an activation step, and an insertion step, which requires the hydrolysis of ATP. The CHLI subunit is the main subunit responsible for the hydrolysis of ATP in the process of enzymatic reactions (Jensen et al., 1999; Reid et al., 2003) and is essential for maintaining the CHLI-CHLD-Mg-ATP complex (Lake et al., 2004; Luo et al., 2013). It is the only reported subunit modulated by redox regulation *via* the chloroplast thioredoxin system (Ikegami et al., 2007; Luo et al., 2012; Perez-Ruiz et al., 2014). In addition, it is also involved in chloroplast reactive oxygen species homeostasis and Ca^2+^ signaling in pea (Luo et al., 2016). As a key regulatory point of chlorophyll biosynthesis, the CHLI subunit might be the primary target to engineer at a molecular level. The CHLI subunit belongs to the ATPase associated with various cellular activities (AAA+) superfamily^13,14^. It possesses characteristic motifs involved in ATP binding such as Walker A and B (W-A and W-B) motifs, sensors 1 and 2 (S-1 and S-2) motifs, presensor I and II (PS-I and II) insert, and arginine finger (ARG-finger) motif (Reid et al., 2003; Lake et al., 2004). It is reported that the mutations in PS-II insert and S-2 motif as well as the mutations between the S-1 motif and the ARG-finger motif could abolish the function of Mg-chelatase in plant (Zhang et al., 2006; Campbell et al., 2014; Gao et al., 2016; Du et al., 2018; Mao et al., 2018). However, the peptide fragments of the CHLI responsible for protein-protein interaction and ATPase activity are unclear.

Interestingly, sequence data in the National Center for Biotechnology Information (NCBI) database^1^ revealed that there is more than one *CHLI* gene in the genomes of most dicots and some algae (Rissler et al., 2002; Huang and Li, 2009; Brzezowski et al., 2016; Sawicki et al., 2017; Zhang et al., 2018). However, the functions of different *CHLI* genes have been only studied in *Chlamydomonas reinhardtii* and *Arabidopsis thaliana*, and conflicting results have been reported (Rissler et al., 2002; Apchelimov et al., 2007; Kobayashi et al., 2008; Huang and Li, 2009; Brzezowski et al., 2016; Sawicki et al., 2017). Rissler et al. (2002) reported that Arabidopsis AtCHLI2 supports only limited chlorophyll synthesis and Apchelimov et al. (2007) proposed that AtCHLI2 is not functional in the Mg-chelatase complex, while Kobayashi et al. (2008) revealed that AtCHLI2 contributes to the assembly of the Mg-chelatase complex and Huang et al. showed that AtCHLI2 can substitute for AtCHLI1 (Huang and Li, 2009). In *C*. *reinhardtii*, Brzezowski et al. (2016) found that CrCHLI2 cannot substitute for CrCHLI1, but Sawicki et al. (2017) demonstrated that CrCHLI2 stimulates Mg-chelatase activity in chlorophyll synthesis. In our previous study, the whole genome data of pea were not available at that time, thus, *CHLI* was silenced in pea by virus-induced gene silencing (VIGS) using a partial *CHLI* sequence obtained by a homology-based cloning method, demonstrating the essential role of pea CHLI in Mg-chelatase activity and chlorophyll biosynthesis (Luo et al., 2013). By searching the pea genome-wide database released in 2019^2^ (Kreplak et al., 2019), the sequence used for silencing pea *CHLI* in our previous study (Luo et al., 2013) matched two different genes (Psat1g200160 and Psat7g118680), suggesting that there are two *CHLI* genes in pea and both of them were silenced in our previous study (Luo et al., 2013). To further explore whether these two genes encoded CHLI subunits and what roles they played in chlorophyll biosynthesis, the present study characterized them at transcriptional and protein levels and identified the peptide fragments of CHLI responsible for protein-protein interactions and enzyme activity, which may provide the primary targets to improve photosynthetic efficiency in pea.

## Materials and Methods

### Plant Material and Growth Conditions

Pea (*P. sativum* ‘Torsdag’; JI992) seeds were cleaned and soaked in tap water for 24 h and germinated on moist filter paper in the dark for 2 days before being planted in soil. Plants were grown in growth chambers (22°C, 65% relative humidity, 250 μmol m^–2^ s^–1^, 16/8 h light/dark photoperiod).

### Cloning of PsCHLI cDNA Sequences

A λ*gt11* cDNA library, representing the leaf mRNA of a 7-day-old pea (Clontech Laboratories, Inc., Mountain View, CA, United States), was screened using polymerase chain reaction (PCR) as previously described (Alfandari and Darribère, 1994), with primers (Ls-PsCHLI-F/R, Supplementary Table 1) designed according to the conserved sequence of the *CHLI* genes in the legume family. The inserted fragments in the PCR-positive clones were characterized by DNA sequencing with the λgt11 insertion checking primers (ICP-F/R, Supplementary Table 1) and were screened by the Basic Local Alignment Search Tool^3^ to confirm whether the clones contained *PsCHLIs*. The positive clones containing *PsCHLIs* were assembled by sequence similarity to obtain the longest sequences for *PsCHLIs*, which were finally verified by reverse transcription-PCR (RT-PCR) from JI992 leaf tissues and DNA sequencing with checking and sequencing primers for *PsCHLIs* (PsCHLI1-F/R and PsCHLI2-F/R, Supplementary Table 1).

### Phylogenetic Analysis

Amino acid sequences of the Mg-chelatase CHLI subunit homologs were obtained from the NCBI database (see text footnote 1), aligned using ClustalX software v2.1,^4^ and refined using GeneDoc software v2.7.^5^ A neighbor-joining (NJ) tree was constructed based on 1,000 bootstrap replications using MEGA 6.0.^6^

### Promoter Analysis

The 1,500 bp region upstream of the translation start codon ATG of *PsCHLI1* and *PsCHLI2* was obtained from *P. sativum* v1a banks and the 1,500 bp region upstream of the translation start codon ATG of Arabidopsis *AtCHLI1* and *AtCHLI2* was obtained from NCBI database. The sequences were analyzed to study the regulatory elements in the promoter regions of each gene using the online program PlantCARE^7^ (Lescot et al., 2002). Since the CAAT box is a proximal promoter element, the predicted CAAT boxes were counted in the 400 bp region upstream of the genes.

### Gene Expression Analysis

For examination of the expression of *PsCHLI1* and *PsCHLI2* in different organs, RNA was extracted from organs at different developmental stages. The roots, leaves, and stems were harvested from 1-week-old seedlings. The flowers were sampled from flowering plants (45 days old). Young pods (3–4 cm long) and immature seeds were sampled from 60-day-old plants. Each organ sample was collected from at least four plants and mixed for RNA isolation. Three biological replicates were performed.

Sterilized pea seeds were germinated on moist filter paper in the dark for 2 days and then transferred into growth chambers (22°C, 65% relative humidity, 250 μmol m^–2^ s^–1^, 16/8 h light (8:00–24:00)/dark photoperiod) for 7 days. Then, the leaves were collected from the seedlings at different time points [8:00 (light on), 12:00, 16:00, 20:00, 24:00 (light off), 4:00] to examine the expression of *PsCHLI1* and *PsCHLI2* under the diurnal changes. For each time point, leaves from at least four plants were collected and mixed for RNA isolation at once, and three biological replicates were performed. For sample collection during dark hours, samples were immediately collected without exposure to light.

Pea seedlings were grown in the dark for 7 days and illuminated for 2, 4, 8, 16, and 24 h. For each irradiation time, leaves from at least four plants were collected and mixed for RNA isolation at once, and three biological replicates were performed. RNA was extracted for examining the expression of *PsCHLI1* and *PsCHLI2* in response to light.

To examine the expression of *PsCHLI1* and *PsCHLI2* following hormone and stress treatments, sterilized pea seeds were germinated on moist filter paper in the dark for 2 days and then transferred into growth chambers (22°C, 65% relative humidity, 250 μmol m^–2^ s^–1^, 16/8 h light/dark photoperiod) for 7 days. The seedlings were then transferred into ABA (0.1 mM), NaCl (300 mM), and PEG6000 (20%, w/v) solutions for 24 h at 22°C. For heat treatment, the seedlings were grown in water for 24 h at 37°C. Seedlings grown in water at 22°C were used as a control. For each treatment, leaves from at least four plants were collected and mixed for RNA isolation at once, and three biological replicates were performed.

Furthermore, RNA was extracted from the top and premature leaves of VIGS plants at 14 days after infiltration (dpi) at 12:00.

The total RNA was extracted from 20 to 40 mg of samples using TRIzol^®^ Reagent (Thermo Fisher Scientific, Waltham, MA, United States) according to the manufacturer’s instructions. Quantitative real-time (qPCR) was performed as previously described (Luo et al., 2013). The primers used in the qPCR amplification are described in Supplementary Table 1. The 2^–ΔΔ*Ct*^ method was used to calculate relative expression. The transcript levels were quantitatively normalized to the transcript level of pea *EF-1*α (GenBank: X96555), which encodes the elongation factor 1-α.

The transcriptional level between *PsCHLI1* and *PsCHLI2* was also compared by RNA-seq using pea leaves. Each leaf sample was collected from at least four 1-week-old plants and mixed for RNA isolation at once. Three biological replicates were performed. For each biological replicate, 1 μg leaf total RNA was used to generate sequencing libraries by NEBNext^®^ Ultra™ II RNA Library Prep Kit for Illumina^®^ (New England Biolabs Inc., Ipswich, MA, United States) following the manufacturer’s recommendations. The library preparations were sequenced on an Illumina platform, and paired-end reads were generated. The raw reads were further processed with an online bioinformatic pipeline tool, BMKCloud.^8^ Raw data (raw reads) in the fastq format was first processed through in-house perl scripts and then adapter, ploy-N, and low-quality reads were removed to obtain clean data (clean reads). The clean reads were then mapped to the pea genome sequence using HISAT2 v2.2.1 software^9^ (Kim et al., 2019). Only reads with a perfect match or one mismatch were further analyzed and annotated based on the pea genome. Gene expression levels were estimated by fragments per kilobase of transcript per million fragments mapped.

### Subcellular Localization

The coding sequences of *PsCHLI1* and *PsCHLI2* were synthesized through DNA synthesis (Genscript Biotech Corporation, Nanjing, China) and cloned into the pM999 vector (C-terminal fused YFP). The resulting plasmids (pM999-PsCHLI1 and pM999-PsCHLI2) and pM999 empty vector were transformed into pea protoplasts according to our previously reported method (Luo et al., 2018). The transformed pea protoplasts were incubated in the dark for 24 h, and images were captured with a laser confocal microscope (TCS SP2; Leica).

### Virus-Induced Gene Silencing Assay

The 1–184 bp and 1,358–1,504 bp *PsCHLI1* cDNA and the 1–99 bp and 1,297–1,577 bp *PsCHLI2* cDNA sequences were synthesized through DNA synthesis (Genscript Biotech Corporation) and inserted into a VIGS vector, pCAPE2, resulting in the plasmids: pCAPE2–PsCHLI1 and pCAPE2–PsCHLI2 (Supplementary Figure 1). The VIGS assay was performed as follows: the pCAPE1 plasmid was co-inoculated with pCAPE2–PsCHLI1, pCAPE2–PsCHLI2, the previously constructed pCAPE2-PsCHLI (Luo et al., 2013), and pCAPE2-GFP (negative control) (Luo et al., 2013) into *P. sativum* (cv. Torsdag; JI992) plants through *Agrobacterium* infiltration. The infected plants were grown in growth chambers (22°C, 65% relative humidity, 250 μmol m^–2^ s^–1^, 16/8 h light/dark photoperiod).

### Determination of Chlorophyll Content

The total chlorophyll (chlorophyll, a + b) was extracted with 100% acetone, and the concentration was determined spectrophotometrically according to our previous study (Luo et al., 2016).

### Construction of the Plasmids for Y2H Assay, Glutathione S Transferase Pull-Down Assay, and Prokaryotic Expression

The cDNA fragments encoding PsCHLI1N, PsCHLI1M, PsCHLI1C, PsCHLI1N plus PsCHLI1M (PsCHLI1NM), PsCHLI1M plus PsCHLI1C (PsCHLI1MC), and PsCHLI1N plus PsCHLI1M plus PsCHLI1C (PsCHLI1NMC), as well as PsCHLI2, and the PsCHLD subunit minus the cTPs, were generated through RT-PCR using the primers described in Supplementary Table 1. The cDNA fragments were then inserted into the prey vector, pGADT7, bait vector, pGBKT7, and prokaryotic expression vectors, pGEX6P-1 (GST tag) and pET-28a (6 × histidine tag, His tag), without disturbing the open reading frame, resulting in the following plasmids: pGADT7-PsCHLI1N, pGADT7-PsCHLI1M, pGADT7-PsCHLI1C, pGADT7-PsCHLI1NM, pGADT7-PsCHLI1MC, pGADT7-PsCHLI1NMC, pGADT7-PsCHLI2, pGEX6P-1-PsCHLI1N, pGEX6P-1- PsCHLI1M, pGEX6P-1-PsCHLI1C, pGEX6P-1-PsCHLI1NM, pGEX6P-1-PsCHLI1MC, pGEX6P-1-PsCHLI1NMC, pGBKT7-PsCHLI1NMC, pGBKT7-PsCHLI2, pET-28a-PsCHLI1NMC, pET-28a-PsCHLI2, pGBKT7-PsCHLD, and pET-28a-PsCHLD.

### Y2H Assay

*Saccharomyces cerevisiae* strain AH109 was co-transformed with both bait and prey plasmids using the lithium acetate method according to the manufacturer’s protocol (Clontech, protocol PT3247-1). The transformants were first selected on Synthetic Dropout (SD/-Leu/-Trp) and scraped onto high-stringency quadruple-dropout media (SD/-Leu/-Trp/-His/-Ade) supplemented with 40 mg/L X-a-Gal to screen the interactions.

### Glutathione S Transferase Pull-Down Assay

*Escherichia coli* strain BL21 was co-transformed with both GST-tagged and His-tagged prokaryotic expression plasmids by electroporation. The expression of recombinant proteins was performed according to our previously published paper (Luo et al., 2018). The GST tag fused proteins and their interacting proteins in the cell lysates were pulled down by glutathione agarose (Thermo Fisher Scientific, Waltham, MA, United States), and detected by western blot using an anti-His tag antibody (Abcam PLC, Cambridge, MA, United States) and an anti-CHLD antibody (Luo et al., 2013).

### Measurement of ATPase and Mg-Chelatase Activity

The expression of GST-tagged prokaryotic expression plasmids was performed as described previously (Luo et al., 2018). The recombinant proteins were purified using glutathione agarose (Thermo Fisher Scientific), and the GST tag was removed by PreScission Protease (Thermo Fisher Scientific) according to the user manual. The ATPase activity of recombinant PsCHLI1N, PsCHLI1M, PsCHLI1C, PsCHLI1NM, PsCHLI1MC, and PsCHLI1NMC was measured as previously described (Luo et al., 2012). The activity of Mg-chelatase reconstituted by the different domains of the pea CHLI subunit and the recombinant rice CHLD, CHLH, and GUN4 proteins, expressed and purified using the previously published plasmids (Zhou et al., 2012), was performed as previously described (Luo et al., 2018). Mg-chelatase activity in the VIGS plants was determined by a stopped fluorometric assay as described by Guo et al. (1998).

### Statistical Analyses

Data are expressed as the means ± S.E.M. The differences between the controls and samples were assessed with one-way analysis of variance and the Dunnett’s test. Statistically significant differences were determined at *P* < 0.05 by the statistical software GraphPad Prism (version 5.01).

## Results

### Two CHLI Subunits Were Identified in Pea

cDNA encoding the CHLI subunit was identified by PCR-based screening of the pea leaf λ*gt11* cDNA library with primers designed according to the conserved sequence of the *CHLI* coding sequence in the legume family. Two different cDNAs were identified and verified by reverse transcription-polymerase chain reaction from JI992 leaf tissues and DNA sequencing. The 1,513-bp cDNA was named *PsCHLI1* (GenBank: JN198382), which contained a 104-bp-long 5′-untranslated region, 140-bp-long 3′-untranslated region, and 1,269-bp-long coding sequence. The 1,577-bp cDNA was named *PsCHLI2* (GenBank: MN128704), containing a 41-bp-long 5′-untranslated region, 282-bp-long 3′-untranslated region, and a 1,254-bp-long coding sequence.

After searching the pea genome-wide database (see text footnote 2) for *PsCHLI1* and *PsCHLI2* cDNA, the *PsCHLI1* and *PsCHLI2* genes were found to be located on chromosomes 1 (Psat1g200160.1, chr1LG6: 350733206-350736270) and 7 (Psat7g118680.1, chr7LG7: 195953907-195956035), respectively (Supplementary Figure 2). Both *PsCHLI* genes contained three exons and two introns and same lengths of exon 2 and the coding sequence in exon 3 (Supplementary Figure 2). *PsCHLI1* had longer introns and five-prime (5′) and three-prime (3′) untranslated regions than *PsCHLI2* (Supplementary Figure 2).

Both *PsCHLI1* and *PsCHLI2* encode ∼46 kDa polypeptides with 422 and 417 amino acid residues (aa), respectively, which are similar in molecular mass to their corresponding homologs identified in other plant species (Supplementary Figure 3). PsCHLI1 and PsCHLI2 are highly similar, sharing 82% amino acid identity (Supplementary Figure 3). Alignment of the PsCHLIs to their orthologs from different species showed that the numbering of secondary structure elements in PsCHLIs follows the convention for AAA + proteins containing Walker A and B motifs (W-A and W-B). W-A, W-B, presensor I (PS-I) insert, helix2 insert (H2-insert), arginine finger (ARG-finger), and sensors 1 and 2 (S-1 and S-2) were conserved in PsCHLI1 and PsCHLI2 (Supplementary Figure 3).

### PsCHLI1 and PsCHLI2 Were Derived From an Ancient Duplication in Legumes

**FIGURE 1:**
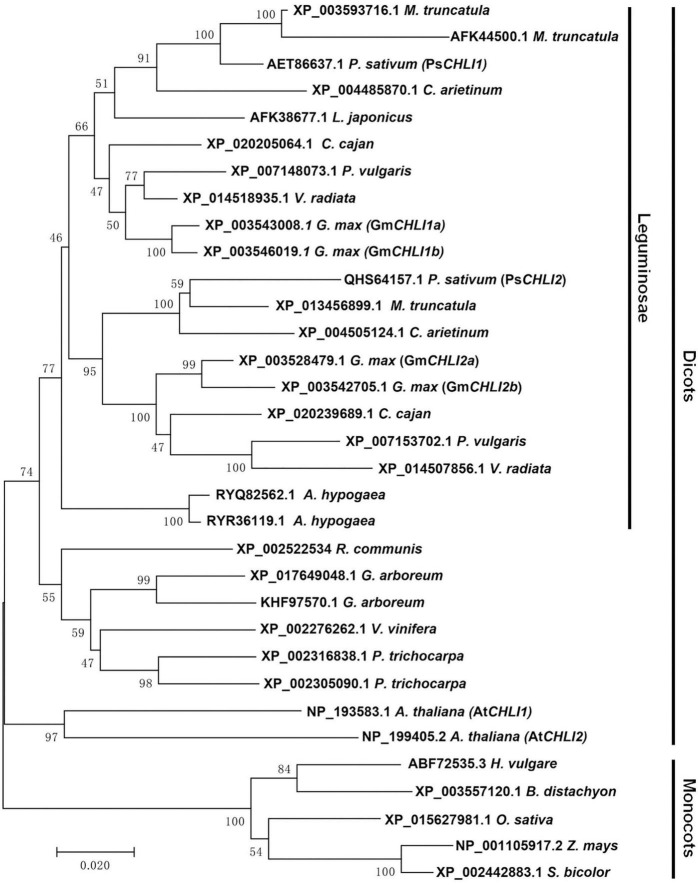
Phylogenetic analysis of plant CHLI subunits. Using the neighbor-joining (NJ) method, a bootstrap consensus tree was constructed on the base of 33 aligned CHLI amino acid sequences from 14 dicotyledons to 5 monocotyledons.

Using the neighbor-joining (NJ) method, a bootstrap consensus tree was constructed on the basis of 33 aligned CHLI amino acid sequences from 19 higher plant species, comprising 14 dicotyledons (*P. sativum*, *Glycine max*, *Phaseolus vulgaris*, *Vigna radiata*, *Cajanus cajan*, *Medicago truncatula*, *Cicer arietinum*, *Arachis hypogaea*, *Lotus japonicus*, *Ricinus communis*, *Gossypium arboreum*, *Vitis vinifera*, *Populus trichocarpa*, and *A. thaliana*) and 5 monocotyledons (*Hordeum vulgare*, *Brachypodium distachyon*, *Oryza sativa*, *Z. mays*, and *Sorghum bicolor*) to investigate the evolution history of the plant CHLI subunit. The sequences of CHLIs were clustered into dicot and monocot clades and subsequently diverged by family (Figure 1). The legume CHLI homologs were sub-grouped into two clades. One clade contains *A. hypogea* which encodes two recently duplicated CHLIs (Figure 1). Another clade contains *P. sativum*, *Glycine max*, *Phaseolus vulgaris*, *Vigna radiata*, *Cajanus cajan*, *Medicago truncatula*, *Cicer arietinum*, and *Lotus japonicus* (Figure 1). Interestingly, the same species in this clade were divided into two subclades except *L. japonicus* that encodes only one CHLI (Figure 1), indicating that *CHLIs* in *P. sativum*, *Glycine max*, *Phaseolus vulgaris*, *Vigna radiata*, *Cajanus cajan*, *Medicago truncatula*, and *Cicer arietinum* were derived from an ancient duplication and maintenance of the duplicated genes. In addition, *CHLIs* in *Glycine max* and *Medicago truncatula* apparently experienced the recent duplications (Figure 1).

### Regulatory Elements in the Promoter Regions of PsCHLI1 and PsCHLI2

More regulatory elements in promoter regions were predicted in the upstream region of *PsCHLI1* compared with that of *PsCHLI2* (54 vs. 29, Supplementary Table 2), especially for the CAAT box (13 vs. 2, Supplementary Table 2). Among *cis*-acting regulatory motifs, 12 and 7 light responsive elements, 21 and 10 hormone responsive elements, and 8 and 10 stress responsive elements were predicted in the promoter regions of *PsCHLI1* and *PsCHLI2*, respectively (Supplementary Table 2).

### PsCHLI1 and PsCHLI2 Show Different Expression Profiles

**FIGURE 2:**
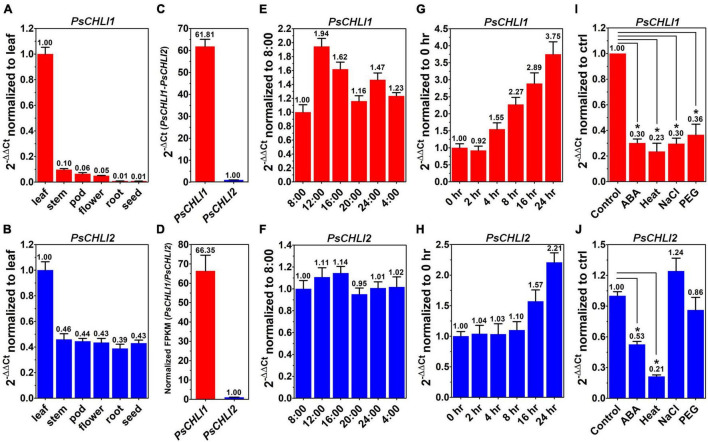
Expression profiles of *PsCHLI1* and *PsCHLI2*. The expression of *PsCHLI1* and *PsCHLI2* in different organs **(A,B)**, time points **(E,F)**, irradiation times **(G,H)**, abscisic acid (ABA, **I,J**), and abiotic stresses (heat, NaCl, and PEG6000, **I,J**) were examined by quantitative real-time PCR (qPCR). The expression level of *PsCHLI1* and *PsCHLI2* was compared by qPCR **(C)** and RNA sequencing **(D)**. The 2^–ΔΔ*Ct*^ method was used to calculate the relative expression for qPCR. The transcript levels were quantitatively normalized to the transcript level of pea *EF-1*α. Gene expression levels for RNA sequencing were estimated by fragments per kilobase of transcript per million fragments mapped (FPKM). Error bars indicate the standard error of the mean (SEM) from three biological replicates, *P < 0.05, one-way analysis of variance.

To study the role of PsCHLIs in chlorophyll biosynthesis, the expression profiles of their encoding genes were examined through qPCR. The amplification efficiencies of the primers for *PsCHLI1*, *PsCHLI2*, and *EF-1*α (reference gene) were 105.62, 107.77, and 99.52%, respectively (Supplementary Figure 4), indicating that the primers were effective for qPCR. The transcription level of *PsCHLI1* and *PsCHLI2* was investigated in roots, stems, leaves, flowers, pods, and immature seeds. The results revealed that *PsCHLI1* was most highly expressed in leaves but at a low level in stems, flowers, and pods, and an almost negligible level in roots and immature seeds (Figure 2A). The expression level of *PsCHLI2* was about twofold higher in leaves than in other organs (Figure 2B).

Since the amplification efficiencies of the primers for *PsCHLI1* and *PsCHLI2* were quite similar (Supplementary Figure 4), the expression level of *PsCHLI1* and *PsCHLI2* were compared by qPCR. *PsCHLI1* was more highly expressed than *PsCHLI2* in leaves (61.81 vs. 1.00, Figure 2C). This was confirmed by RNA sequencing (RNA-seq) using pea leaves (Figure 2D and Supplementary Table 3).

In addition, the transcription level of *PsCHLI1* showed diurnal changes (Figures 2E,F). When the light was on at 8:00, *PsCHLI1* was expressed at the lowest level and then its transcripts reached the highest level at 12:00, decreased at 16:00, returned to an equivalent level as that of 8:00 at 20:00, and increased at 24:00 when the light was off (Figure 2E). Subsequently, *PsCHLI1* was downregulated at 4:00 and to the lowest level at 8:00 (Figure 2E). In contrast, expression of *PsCHLI2* was relatively steady in different time points (Figure 2F). The expression profiles of *PsCHLI1* and *PsCHLI2* responding to light were also examined. The results showed that the expression of *PsCHLI1* was upregulated gradually from 4 to 24 h illumination (Figure 2G), while the expression of *PsCHLI2* was upregulated at 16 and 24 h illumination (Figure 2H).

We also examined the expression of *PsCHLI1* and *PsCHLI2* responding to abscisic acid (ABA) and abiotic stresses (heat, salt, and drought) by the qPCR. The results showed that *PsCHLI1* was significantly downregulated following ABA, heat, salt, and drought treatments (*P* < 0.05, Figure 2I) and *PsCHLI2* was only significantly downregulated following ABA and heat treatments (*P* < 0.05, Figure 2J).

### PsCHLI1 and PsCHLI2 Are Both Located in Pea Chloroplasts

**FIGURE 3:**
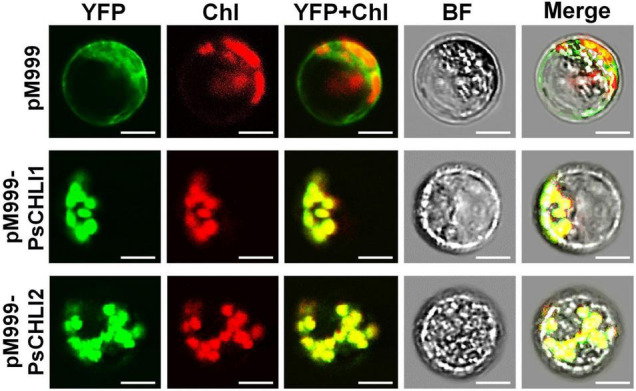
Subcellular localization of PsCHLI1 and PsCHLI2. PsCHLI1 and PsCHLI2 tagged with a C-terminal yellow fluorescent protein (YFP) were transiently expressed under the control of the 35S promoter in pea protoplasts, and images were captured with a laser confocal microscope (TCS SP2; Leica). Chl, chlorophyll auto-fluorescence, BF, bright field. Scale bar = 20 μm.

The putative chloroplast transit peptides (cTPs) were predicted at the N-terminus of PsCHLI1 (1-62 aa) and PsCHLI2 (1-34 aa) by ChloroP v1.1^10^ (Supplementary Figure 3). To confirm their subcellular localization, yellow fluorescent fusion proteins were generated by fusing yellow fluorescent protein (YFP) to the C terminus of full-length PsCHLI1 and PsCHLI2. These fusion proteins and the YFP were transiently expressed in pea leaf protoplasts and observed by confocal laser scanning microscopy. The results showed that YFP fluorescence was only visualized in the chloroplasts of the protoplasts that transiently expressed YFP-fused PsCHLI1 and PsCHLI2 (Figure 3), indicating that PsCHLI1 and PsCHLI2 are both located in pea chloroplasts.

### PsCHLI1, but Not PsCHLI2, Is Essential for Chlorophyll Biosynthesis in Pea

**FIGURE 4:**
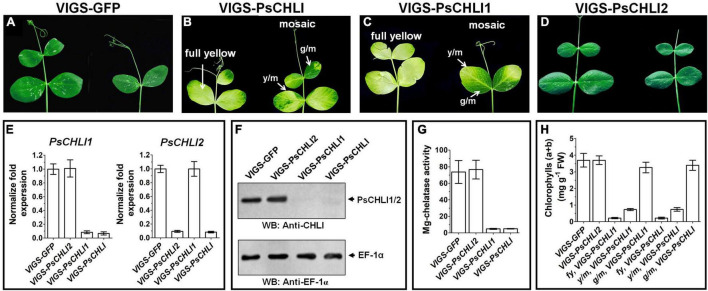
Phenotypes of virus-induced gene silencing (VIGS) plants. *PsCHLI1* and *PsCHLI2* were silenced in pea by a VIGS method. VIGS-GFP plants represented a negative control for the effect of virus infection **(A)**. VIGS-PsCHLI plants, described in our previous study (Luo et al., 2013), were used as a positive control **(B)**. VIGS-PsCHLI1 plants showed yellow leaves. Three types of leaf phenotypes are shown: fully yellow leaves; y/m, yellow sectors from mosaic leaves; g/m, green sectors from mosaic leaves **(C)**. Leaves of VIGS-PsCHLI2 plants resembled those in VIGS-GFP control plants **(D)**. The expression of *PsCHLI1* and *PsCHLI2* in VIGS plants was measured by quantitative real-time PCR. The 2^–ΔΔ*Ct*^ method was used to calculate the relative expression. The transcript levels were quantitatively normalized to the transcript level of pea *EF-1*α **(E)**. The protein level of PsCHLI1 and PsCHLI2 in VIGS plants was measured by western blot. EF-1α was used as a loading control **(F)**. Mg-chelatase activity **(G)** and chlorophyll content **(H)** were also examined in VIGS plants. All plants had three independent infiltrations and were observed 3 weeks after infiltration. Error bars indicate the SEM from at least six VIGS plants in three independent infiltrations.

Although PsCHLI1 and PsCHLI2 have high sequence similarity and are both located in pea chloroplasts, their expression profiles suggest different roles for the PsCHLIs in chlorophyll biosynthesis. To study the roles of PsCHLI1 and PsCHLI2 in chlorophyll biosynthesis, the 5′- and 3′-end sequences of *PsCHLI1* and *PsCHLI2*, which have low identity, were selected as the targets for a VIGS assay to specifically silence *PsCHLI1* and *PsCHLI2* in pea (Supplementary Figure 1). As a control, the VIGS-GFP and VIGS-PsCHLI plants described in our previous study (Luo et al., 2013) were used (Figures 4A,B and Supplementary Figure 5). The phenotype of *PsCHLI1* silenced (VIGS-PsCHLI1) plants resembled VIGS-PsCHLI plants (in which both *PsCHLI1* and *PsCHLI2* were targeted for silencing), demonstrating three types of leaves, including fully yellow leaves (fy), yellow sectors from mosaic leaves (y/m), and green sectors from mosaic leaves (g/m) (Figures 4B,C

and Supplementary Figure 5), while *PsCHLI2* silenced (VIGS-PsCHLI2) plants showed no phenotype compared with VIGS-GFP plants (Figure 4D and Supplementary Figure 5). *PsCHLI1* in VIGS-PsCHLI1 plants and *PsCHLI2* in VIGS-PsCHLI2 plants, as well as *PsCHLI1* and *PsCHLI2* in VIGS-PsCHLI plants, were silenced by more than 90% (Figure 4E), while neither silencing *PsCHLI1* nor *PsCHLI2* changed the transcriptional level of the other *PsCHLI* homologs (Figure 4E). In addition, to examine the protein level of PsCHLI1 and PsCHLI2 in the VIGS plants, western blot was performed using a previously verified anti-Arabidopsis CHLI1 antibody (Luo et al., 2013). This antibody recognized both the recombinant PsCHLI1 and PsCHLI2 and overexpressed them in *E. coli* due to the high similarity in amino acids between PsCHLI1 and PsCHLI2 (Supplementary Figure 6). The target bands that showed equivalent intensity and matched the predicted molecular weight of PsCHLIs were detected in VIGS-GFP and VIGS-PsCHLI2 plants (Figure 4F). In contrast, neither PsCHLI1 nor PsCHLI2 were detected in the leaves of both VIGS-PsCHLI1 and VIGS-PsCHLI plants (Figure 4F). Furthermore, Mg-chelatase activity and chlorophyll content were hardly detectable in the yellow leaf of both VIGS-PsCHLI and VIGS-PsCHLI1 plants, while they were not affected in green leaves of VIGS-PsCHLI2 plants compared with VIGS-*GFP* control plants (Figures 4G,H). The development of flowers, pods, and seeds was not affected in either VIGS-PsCHLI1 or VIGS-PsCHLI2 plants, which was consistent with our reported phenotype for VIGS-PsCHLI plants (Luo et al., 2013). The yield of pods and seeds from the VIGS-PsCHLI2 plant is equivalent to that in VIGS-GFP plants. However, both VIGS-PsCHLI and VIGS-PsCHLI1 plants died earlier than VIGS-GFP plants, and thus fewer pods and seeds were harvested from both VIGS-PsCHLI and VIGS-PsCHLI1 plants than VIGS-GFP plants.

### The N-Terminal Fragment of PsCHLI1 Mediates the Formation of PsCHLI1 Dimers, and the Middle Fragment of PsCHLI1 Is Involved in the Interaction With PsCHLD

**FIGURE 5:**
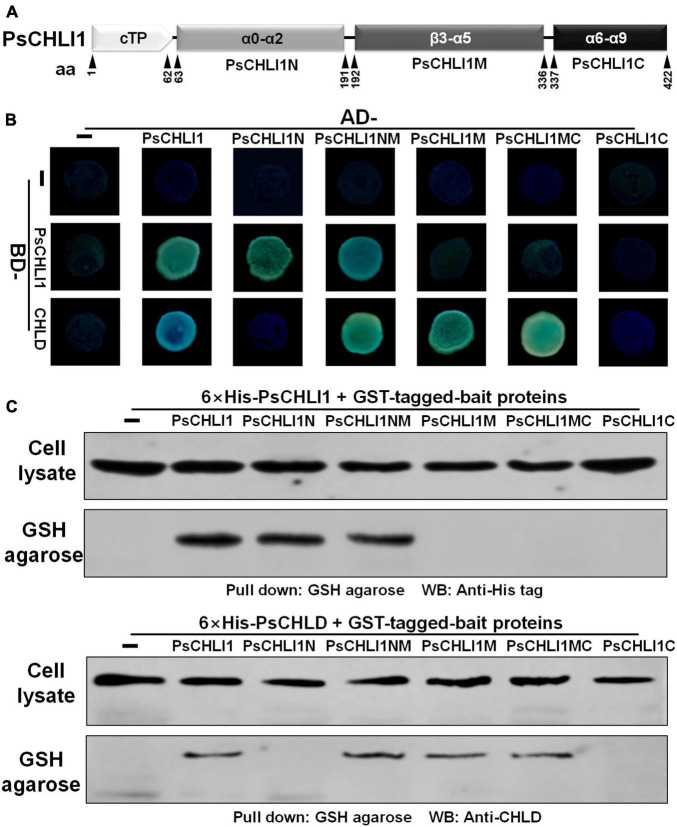
Peptide fragments of PsCHLI1 for protein-protein interactions. **(A)** Four peptide fragments of PsCHLI1. The amino acid positions (aa) are indicated below the elements. Secondary structure elements in the different peptide fragments are shown in the boxes. α, α-helices; β, β-sheets. The interactions between PsCHLI1 or PsCHLD and the different fragments of PsCHLI1 were determined by the yeast two-hybrid assay **(B)** and glutathione S transferase (GST) pull-down assay **(C)**. cTP, chloroplast transit peptide; PsCHLI1N, the N-terminal fragment of PsCHLI1; PsCHLI1M, the middle fragment of PsCHLI1; PsCHLI1C, the C-terminal fragment of PsCHLI1; PsCHLI1NM, PsCHLI1N fused with PsCHLI1M; PsCHLI1MC, PsCHLI1M fused with PsCHLI1C; GSH, glutathione.

Previous studies have demonstrated that the interactions between subunits are essential for maintaining Mg-chelatase activity (Masuda, 2008). The present study showed that PsCHLI1 is the predominant CHLI subunit in pea leaves and is essential for chlorophyll biosynthesis in pea. Therefore, the role of PsCHLI1 in subunit-subunit interactions was further explored. According to the structure-based alignment of the amino acid sequences of CHLI/BchI (Supplementary Figure 3), PsCHLI1 (minus the chloroplast transit peptide) was characterized into three peptide fragments: the N-terminal fragment (PsCHLI1N, Val63 to Cys191), containing an ATP/GTP binding motif A (Walker A) and an α1-β2-β hairpin motif; the C-terminal fragment (PsCHLI1C, Ser337 to Ser422), possessing four α helixes and the sensor 2 region (S2); and the middle fragment (PsCHLI1M, Gly192 to Ser336), linking PsCHLI1N and PsCHLI1C, which includes three insertions into the core AAA topology (H2-insert, PSI-insert and PSII insert), ATP/GTP binding motif B (Walker B), sensor 1 region (S1), and ARG-finger (Figure 5A and Supplementary Figure 3). A yeast two-hybrid (Y2H) assay was performed to explore the role of the above PsCHLI1 fragments in mediating the interactions between PsCHLI1 and PsCHLI1 or PsCHLD. The results showed that PsCHLI1N interacted with PsCHLI1 but not PsCHLD (Figure 5B), while PsCHLI1M interacted with PsCHLD but not PsCHLI1 (Figure 5B). Notably, if PsCHLI1N fused with PsCHLI1M (PsCHLI1NM), homodimerization and an interaction with PsCHLD were both observed (Figure 5B). However, PsCHLI1C did not interact with PsCHLI1 or PsCHLD (Figure 5B). The interaction with PsCHLD was observed only when PsCHLI1C was fused with PsCHLI1M (PsCHLI1MC) (Figure 5B). Furthermore, a glutathione S transferase (GST) pull-down assay was performed to confirm the results of the Y2H assay. For this purpose, GST tag-fused PsCHLI1 was co-expressed with His tag-fused PsCHLI1 or PsCHLD in *E. coli* and pulled down by glutathione agarose. In the negative control, neither His-tagged PsCHLI1 nor PsCHLD were pulled down by GST itself (Figure 5C). His-tagged PsCHLI1 was pulled down by GST-tagged PsCHLI1NMC, PsCHLI1N, and PsCHLI1NM (Figure 5C), and the His-tagged PsCHLD interacted with GST-tagged PsCHLI1NMC, PsCHLI1NM, PsCHLI1M, and PsCHLI1MC (Figure 5C), consistent with the results from the Y2H assay. In addition, whether PsCHLI2 can form homodimers and interact with PsCHLI1 or PsCHLD were also examined by Y2H assay. The result showed that PsCHLI2 can also form homodimer and interact with PsCHLI1 and PsCHLD (Supplementary Figure 7).

### All Three Peptide Fragments of PsCHLI1 Are Essential for Maintaining the Enzyme Activity of PsCHLI1

**FIGURE 6:**
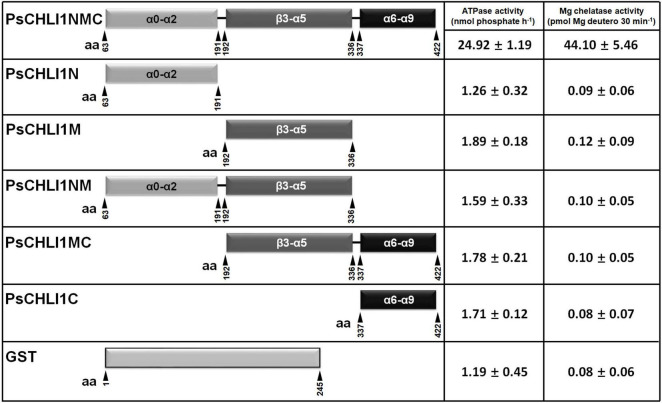
Peptide fragments of PsCHLI1 for enzyme activity. The different fragments of 1 μM PsCHLI1 and GST purified from *E. coli* were used to determine ATPase activity by the Malachite Green colorimetric assay. The different fragments of 0.2 μM PsCHLI1 and GST purified from *E. coli* were combined with recombinant rice CHLD (0.05 μM), CHLH (0.5 μM), and GUN4 (0.5 μM) proteins to reconstitute the Mg-chelatase activity *in vitro* by a stopped fluorometric assay. Data represent the mean ± S.E.M of nine replications from three independent assays. Differences between the GST group and groups of the PsCHLI1 fragments were assessed with analysis of variance. Secondary structure elements in the different peptide fragments are shown in the boxes. α, α-helices; β, β-sheets.

To study the role of the three peptide fragments of PsCHLI1 in maintaining the activities of ATPase and Mg-chelatase, the truncated PsCHLI1 recombinant proteins were purified and expressed in *E. coli* (Supplementary Figure 8). Although PsCHLI1N mediated the formation of the homodimer of the CHLI subunit, it showed no significant ATPase or Mg-chelatase activity compared with the negative control (GST protein itself) (*P* > 0.05, Figure 6). Similar to PsCHLI1N, the action of PsCHLI1M in mediating the interaction between PsCHLI1 and PsCHLD was not sufficient to maintain the activity of ATPase and Mg-chelatase (Figure 6). PsCHLI1C had no ATPase or Mg-chelatase activity since it did not interact with PsCHLI1 and PsCHLD (Figure 6). PsCHLI1NM was also inactive, although this protein could interact with both PsCHLI1 and PsCHLD (Figure 6). Only the combination of the three peptide fragments of PsCHLI1 (PsCHLI1NMC) was essential for maintaining ATPase activity and reconstituting active Mg-chelatase *in vitro* (Figure 6).

## Discussion

### Different Expression Level Between Duplicated *CHLIs* May Be Related to Their Divergent Gene Duplication Pattern

Phylogenetic analysis showed that duplicated *CHLIs* in dicots could be derived from an ancient gene duplication and/or a recent gene duplication (Figure 1). In pea, the duplicated *CHLIs* (*PsCHLI1* and *PsCHLI2*) originated from an ancient duplication (Figure 1) and they differ greatly in their expression level (Figure 2). In Arabidopsis, *AtCHLI1* and *AtCHLI2* were derived from a recent duplication (Figure 1) and *AtCHLI2* was not expressed much lower than *AtCHLI1* (Rissler et al., 2002; Huang and Li, 2009). In addition, the *CHLIs* in soybean experienced both ancient and recent gene duplications, resulting in four *CHLIs* (*GmCHLI1a*, *GmCHLI1b*, *GmCHLI2a*, and *GmCHLI2b*, Figure 1). The two *GmCHLI1* paralogs were more highly expressed than the two *GmCHLI2* paralogs, while there was no obvious difference in expression between *GmCHLI1a* and *GmCHLI1b* or between *GmCHLI2a* and *GmCHLI2b* (Li et al., 2016). These results suggested that the *CHLI* paralogs derived from the ancient gene duplication differ greatly in their expression level, while the expression level of *CHLI* paralogs derived from the recent gene duplication shows no obvious difference. A recent study in soybean indicated that the number of the CAAT box (important for the sufficient transcription of the downstream gene) in proximal promoter of *CHLI* paralogs is positively correlated with their transcription levels (Zhang et al., 2018). This phenomenon is also present in pea and Arabidopsis (Supplementary Table 2). These results implied that the changes of promoter regions in *CHLI* duplicated pairs may affect their expression levels during evolution.

### Only One Copy of Duplicated *PsCHLIs* Acts as Mg-Chelatase CHLI Subunit Due to Non-functionalization at the Expression Level in Pea

Duplicate gene pairs may experience a potential fate that loss-of-function mutations in the coding region and/or the destruction in the regulatory regions of one duplicate led to non-functionalization by losing gene function and/or gene expression (Force et al., 1999). The present study found that *PsCHLI1* was more highly expressed than *PsCHLI2* at both transcriptional and protein levels, suggesting that adequate PsCHLI1 but few PsCHLI2 proteins were present in pea leaves. Although PsCHLI2 could form a homodimer and interacted with PsCHLI1 and PsCHLD and had similar ATPase and reconstituted Mg-chelatase activities to PsCHLI1 *in vitro* (Supplementary Figure 7), the low expression of *PsCHLI2* resulted in few proteins in pea leaves and silencing *PsCHLI2* in pea did not affect leaf Mg-chelatase activity. In addition, pea plants with a silenced *PsCHLI1* had undetectable Mg-chelatase activity in leaves. These results suggested that *PsCHLI2* probably experienced non-functionalization at the expression level and only *PsCHLI1* acted as the key CHLI subunit of Mg-chelatase in pea leaves. Previous studies in Arabidopsis and soybean demonstrated that two copies of duplicated pair of *CHLI* derived from a recent gene duplication showed no or slightly obvious difference at the expression level and participated in chlorophyll synthesis (Huang and Li, 2009; Zhang et al., 2018). In this study, our findings provided a new information about the divergence of *CHLIs* in plants apart from the results in *Arabidopsis* and soybean. To our knowledge, it is the first to investigate the function of a duplicated pair of *CHLI* originating from an ancient gene duplication.

### Roles of PsCHLI1 Motifs in Mg-Chelatase

CHLI is essential for both activation and insertion steps in the enzymatic reaction of Mg-chelatase (Masuda, 2008). In the activation step, six CHLIs are assembled into a hexameric ring structure and interact with the hexameric ring of CHLD to form a CHLIs-CHLDs-Mg-ATP complex for the insertion step, in which CHLI hydrolyzes ATP to provide energy (Lake et al., 2004; Masuda, 2008; Luo et al., 2013). In this study, the PsCHLI2 protein was undetectable in pea leaves by western blot and silencing *PsCHLI2* in pea plants did not affect the Mg-chelatase activity and the chlorophyll content. These results indicated PsCHLI2 is not functional in the Mg-chelatase complex. It is proposed that only PsCHLI1 participates in the assembly of the hexameric CHLI ring structure and interacting with PsCHLD in pea leaves. Therefore, the peptide fragments of the PsCHLI1 responsible for protein-protein interaction and enzyme activity were characterized. The present study found that AAA modules were conserved in PsCHLI1. The Walker A motif in the PsCHLI1N was responsible for CHLI dimerization that is important for the assemble of the ring structure. Three PsCHLI1 homodimers form a hexameric ring structure. The Walker B and sensor 1 motifs in the PsCHLI1M were involved in the interaction between CHLI and CHLD. Although PsCHLI1NM, containing the above motifs, mediated protein interactions, it had no ATPase or Mg-chelatase activity. These results indicated that PsCHLI1C was also required for maintaining enzyme activity, although it was not involved in protein-protein interactions. The sensor 2 motifs (also called sensor arginine, S-2, arginine_361_) located in the PsCHLI1C have been reported to be vital for ATPase activity (Mao et al., 2018). Although the motifs of AAA modules in PsCHLI1 were involved in different steps of enzymatic reaction, they were essential for the function of CHLI. In addition, other motifs in PsCHLI1 may also take part in maintaining Mg-chelatase activity. The point mutations in amino acids, including glycine, arginine, glutamine, and aspartate between the sensor 1 motif and the ARG-finger motif, and arginine in the PS-II insert, could abolish the function of Mg-chelatase in cucumber (Gao et al., 2016), soybean (Campbell et al., 2014; Du et al., 2018), and rice (Zhang et al., 2006). These residues are conserved in PsCHLI1M (glycine_271_, arginine_274_, glutamine_276_, aspartate_279_, and arginine_314_), indicating their roles in the interaction with CHLD. In addition, our previous study showed that cysteines in PsCHLI1N (cysteine_100_ and cysteine_191_) and PsCHLI1C (cysteine_352_ and cysteine_394_) can be redox-regulated and are important for the ATPase activity of CHLI and Mg-chelatase (Luo et al., 2012). Taken together, all AAA modules in PsCHLI1 are essential for maintaining the ATPase activity of CHLI and reconstituting active Mg-chelatase.

## Data Availability Statement

The datasets presented in this study can be found in online repositories. The names of the repository/repositories and accession number(s) can be found below: https://www.ncbi.nlm.nih.gov/, PRJNA752108.

## Author Contributions

C-JW and SL designed the experiments. JZ and TL cloned the pea *CHLI* cDNA and did sequence analyses and performed the yeast two-hybrid assay. JW and JR performed the quantitative real-time PCR. JW constructed the plasmids. C-JW and Y-XZ conducted the subcellular localization. Q-HZ determined the chlorophyll content. JW and L-XX measured the enzyme activity. SL performed the virus-induced gene silencing assay. TL and SL conducted the GST pull-down assay. Y-XY and SL performed phylogeny analysis. X-FX, TL, and SL analyzed the data, interpreted the results, and wrote the manuscript. All authors read and approved the final manuscript.

## Conflict of Interest

The authors declare that the research was conducted in the absence of any commercial or financial relationships that could be construed as a potential conflict of interest.

## Publisher’s Note

All claims expressed in this article are solely those of the authors and do not necessarily represent those of their affiliated organizations, or those of the publisher, the editors and the reviewers. Any product that may be evaluated in this article, or claim that may be made by its manufacturer, is not guaranteed or endorsed by the publisher.

## Footnotes

1
http://www.ncbi.nlm.nih.gov/genbank

2
https://urgi.versailles.inra.fr/blast/

3
https://blast.ncbi.nlm.nih.gov/

4
https://clustalx.software.informer.com/2.1

5
https://genedoc.software.informer.com

6
https://www.megasoftware.net/

7
http://bioinformatics.psb.ugent.be/webtools/plantcare/html/

8
www.biocloud.net

9
https://daehwankimlab.github.io/hisat2/download/

10
http://www.cbs.dtu.dk/services/ChloroP/
