# Supplementary material for: Molecular Characterization of Mg-Chelatase CHLI Subunit in Pea (Pisum sativum L.)
Source: Front Plant Sci. 2022 Jan 25;13:821683. doi: 10.3389/fpls.2022.821683 (PMC8821089; doi:10.3389/fpls.2022.821683)
Supplement: Supplementary file 1 [file Data_Sheet_1.docx]

Supplementary Materials

# Supplementary Table 1. PCR-primers designed for the experiments in this manuscript

| **No.** | **Earmarking** | **Sequence (5'-...- 3') ^a^** | **Application** |
| --- | --- | --- | --- |
| 1 | Ls-PsCHLI-F | CAGGAAAGTCCACAACGGTTAGGTC | PCR screening |
| 2 | Ls-PsCHLI-R | ATCTTCACTCTCAGTTCAGCGTCTC | PCR screening |
| 3 | ICP-F | GGTTTCCATATGGGGATTGGTGG | Insertion checking primer |
| 4 | ICP-R | GACACCAGACCAACTGGTAATGG | Insertion checking primer |
| 5 | PsCHLI1-F | TGATGGTTGGAAACCAATAGAA | PCR checking and sequencing |
| 6 | PsCHLI1-R | GAAGTAAAGGCTTAGAATATCTTG | PCR checking and sequencing |
| 7 | PsCHLI2-F | CAACAACACAAGGAAGAAGC | PCR checking and sequencing |
| 8 | PsCHLI2-R | TGCGTGTAAAAATCATTTCATTC | PCR checking and sequencing |
| 9 | qPsCHLI1-F | CTTTCTTCCCCTTCTTCCAAC | qPCR for *PsCHLI1* |
| 10 | qPsCHLI1-R | AAGGATACACTGGCCTCTGG | qPCR for *PsCHLI1* |
| 11 | qPsCHLI2-F | ACATCACTCTCCCCCTTCCAT | qPCR for *PsCHLI2* |
| 12 | qPsCHLI2-R | CAATCCTCTGAATCTGTTGAGG | qPCR for *PsCHLI2* |
| 13 | qEF-1α-F | GATTGACAGGCGATCTGGTAAGG | qPCR for *EF-1α* gene |
| 14 | qEF-1α-R | GGCTGCTGCTTTGGTGACCTT | qPCR for *EF-1α* gene |
| 15 | *Eco*RI-PsCHLI1N-F | CCG*GAATTC*CGGGTT GCTACCGAAGTTAACTCTGTAG | For *pGEX6P-1-PsCHLI1N*/*-PsCHLI1NM* and *pGADT7-PsCHLI1N*/*-PsCHLI1NM* |
| 16 | *Xho*I-PsCHLI1N-R | CCG*CTCGAG*CGGTCAACAAACCCTATCTTCTGTAGCT | For *pGEX6P-1-PsCHLI1N* and *pGADT7-PsCHLI1N* |
| 17 | *Eco*RI-PsCHLI1M-F | CCG*GAATTC*CgGGGAACAATTGATATCGAAAAAGCTC | For *pGEX6P-1-PsCHLI1M*/*-PsCHLI1MC* and *pGADT7-PsCHLI1M*/*-PsCHLI1MC* |
| 18 | *Xho*I-PsCHLI1M-R | CCG*CTCGAG*CGGTCAAGAAAGTAAACTCCTTGCTGAG | For *pGEX6P-1-PsCHLI1M*/*-PsCHLI1NM* and *pGADT7-PsCHLI1M*/*-PsCHLI1NM* |
| 19 | *Eco*RI-PsCHLI1C-F | CCG*GAATTC*CGGTCAGTTCAAATTGATCAAGATCTGA | For *pGEX6P-1-PsCHLI1C* and *pGADT7-PsCHLI1C* |
| 20 | *Xma*I*-Sal*I-PsCHLI1C-R | TCCC*CCCGGGGTCGAC*GAAGTAAAGGCTTAGAATATCTTG | For *pGEX6P-1-PsCHLI1C*/*-PsCHLI1MC* and *pGADT7-PsCHLI1C*/-*PsCHLI1MC* |
| 21 | *Nde*I-*Eco*RI-PsCHLI1-F | *CATATGGAATTC*CGTCCTGTTGTCAATGTTGCTACCG | For *pGEX6P-1-/pET-28a-/pGADT7-/pGBKT7-PsCHLI1NMC* |
| 22 | *Xma*I*-Sal*I-PsCHLI1-R | *CCCGGGGTCGAC*GTTCTATCTATCTATCACCTTCAGC | For *pGEX6P-1-/pET-28a-/pGADT7-/pGBKT7-PsCHLI1NMC* |
| 23 | *Nde*I-PsCHLI2-F | *CATATG*ACCAAAGGGAAAGTATCTGTGAG | For *pET-28a-PsCHLI2*, *pGADT7-PsCHLI2* and *pGBKT7-PsCHLI2* |
| 24 | *Xho*I-*Pst*I-PsCHLI2-R | *CTCGAGCTGCAG*GCTAGAGAAGACTGGTCATGTTG | For *pET-28a-PsCHLI2*, *pGADT7-PsCHLI2* and *pGBKT7-PsCHLI2* |
| 25 | *Nde*I-PsCHLD-F | *CATATG*CAAAATGGAGCTGTTCTGCAAGC | For *pET-28a-PsCHLD* and *pGBKT7-PsCHLD* |
| 26 | *Bam*HI-PsCHLD-R | *GGATCC*GGTGTCAAGATTGGTTGCTTCT | For *pET-28a-PsCHLD* and *pGBKT7-PsCHLD* |

^a^ Restriction sites are indicated in italic and underlined.

## Supplementary Table 2. The outputs of RNA sequencing

| **Samples** | **Clean reads** | **Clean**  **bases** | **GC Content** | **%≥Q30** | **PsCHLI1** **FPKM** | **PsCHLI2** **FPKM** | **PsCHLI1** **count** | **PsCHLI2** **count** |
| --- | --- | --- | --- | --- | --- | --- | --- | --- |
| Leaf1 | 25,749,559 | 7,718,658,904 | 43.08% | 93.42% | 215.55 | 2.52 | 18570 | 194 |
| Leaf2 | 25,615,710 | 7,669,163,626 | 42.96% | 93.87% | 139.61 | 2.20 | 10702 | 151 |
| Leaf3 | 24,283,990 | 7,276,180,608 | 42.85% | 93.59% | 180.78 | 3.36 | 17948 | 294 |

## Supplementary Table 3. Regulatory elements in the promoter regions of *PsCHLI1*, *PsCHLI2, AtCHLI1* and *AtCHLI2*

| **Motifs** | **Sequence** | ***PsCHLI1*** | ***PsCHLI2*** | ***AtCHLI1*** | ***AtCHLI2*** |
| --- | --- | --- | --- | --- | --- |
| CAAT box | CAAAT/CAAT/  CCAAT | -90, -91, -168, -196, -209,  -239, -244, -275, -304,  -334, -356, -367, -368 | -107, -370 | -37, -38, -72, -73, -245, -382 | -149, -169, -182, -209, -234,  -280, -344, -382, -383 |
| **Subtotal** |  | **13** | **2** | **6** | **9** |
| **Light responsive elements** | | | |  |  |
| ACA-motif | AATTACAGCCATT | -1375 | - |  |  |
| AE-box | AGAAACAA | -795 | -46 | -88 |  |
| AT1-motif | AATTATTTTTTATT | -1375 | - |  | -1011 |
| Box 4 | ATTAAT | -267, -1368, -1387 | -483, -663,  -706, -742,  -1133 |  | -393, -1356 |
| chs-CMA2c | ATATACGTGAAGG | -760 | - |  |  |
| GATA-motif | AAGGATAAGG |  |  | -103 |  |
| G-box | TACGTG/CACGAC/TACGTG | -756, -832, -877 | - | -1033 | -437, -900 |
| I-box | GGATAAGGTG/CCTTATCCT |  |  | -103, -105 | -86 |
| LAMP-element | CCTTATCCA |  |  |  | -84 |
| MRE | AACCTAA | -609 | - |  |  |
| TCT-motif | TCTTAC | -141 | -300 | -390, -544 |  |
| **Subtotal** |  | **12** | **7** | **7** | **7** |
| **Hormone responsive elements** | | | |  |  |
| AAGAA-motif | GTAAAGAAA | -17, -818 | - |  | -808, -1170 |
| ABRE | ACGTG | -756, -877 | - | -437 |  |
| ABRE3a | TACGTG | -756, -877 | - |  | -437 |
| ABRE4 | CACGTA | -756, -877 | - |  | -437 |
| as-1 | TGACG | -955 | - | -476, -521, -808, -882 | -1374 |
| CGTCA-motif | CGTCA | -955 | - | -476, -521, -808, -882 | -1374 |
| ERE | ATTTTAAA | -975, -977, -1231, -1299,  -1337 | -120 |  |  |
| Myc | TCTCTTA | - | -302 |  | -975 |
| MYC | CATTTG/CATGTG | -239, -357, -412, -809,  -1277 | -411, -794,  -816, -1416,  -1437 | -1098, -1106 | -310, -704, -1409 |
| P-box | CCTTTTG | - | -217, -1203 |  |  |
| TGA-element | AACGAC | - | -528 |  | -332, -365 |
| TGACG-motif | TGACG | -955 | - | -476, -521, -808, -882 | -1374 |
| **Subtotal** |  | **21** | **10** | **15** | **13** |
| **Stress responsive elements** | | | |  |  |
| ACTCATCCT sequence | ACTCATCCT | - | -325 |  |  |
| ARE | AAACCA | -94, -371 | -1121, -1254 | -998, -1327 | -227, -267, -272, -616, -877,  -1055 |
| AT-rich sequence | TAAAATACT | -973 | - |  |  |
| LTR | CCGAAA | -614 | -95, -399 |  | -219 |
| Myb-binding site | CAACAG | - | -1154 |  |  |
| MBS | CAACTG | -778 | - | -368, -994 |  |
| MYB | CAACCA/TAACCA | -101, -1018 | - | -663, -908, -1394 | -587 |
| STRE | AGGGG | - | -232, -857,  -958 | -281 |  |
| TC-rich repeats | ATTCTCTAAC | - | -304 |  |  |
| WUN-motif | AAATTACTA | -919 | - |  |  |
| **Subtotal** |  | **8** | **10** | **8** | **8** |
| **Total** |  | **54** | **29** | **36** | **37** |

##
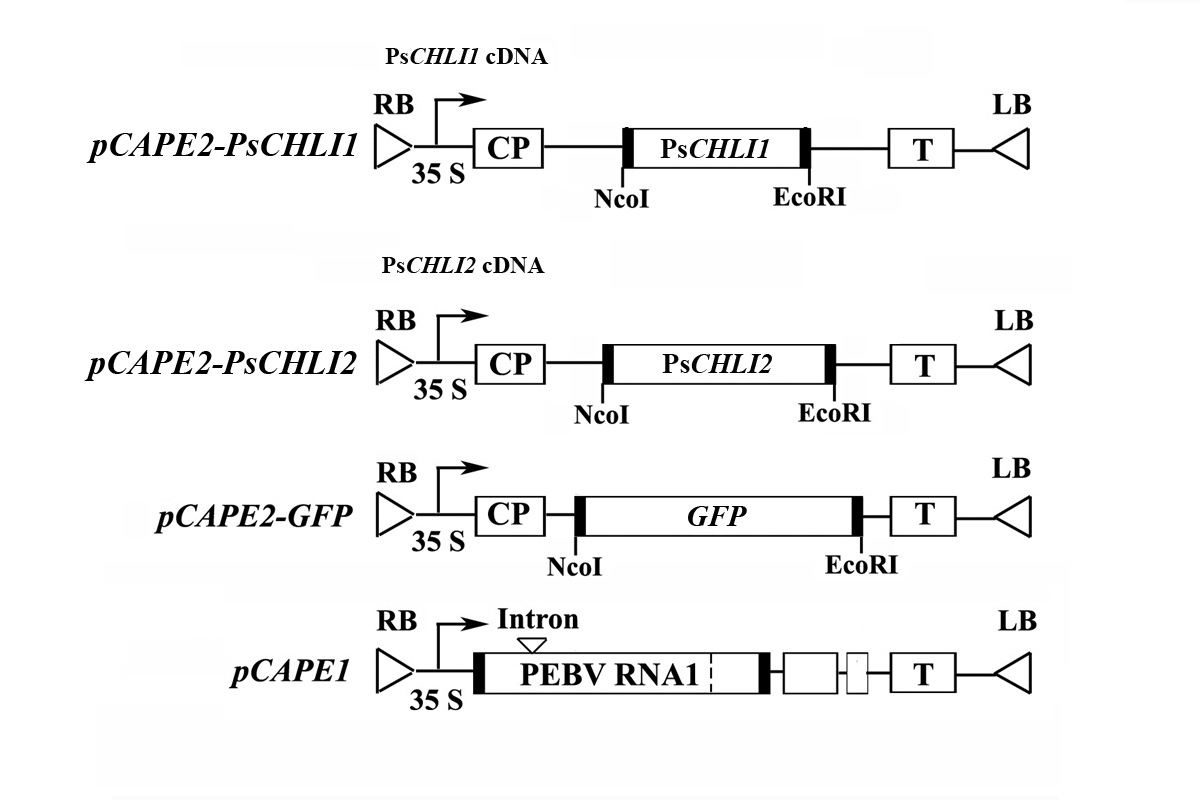
Supplementary Figure 1. Pea VIGS binary vectors based on PEBV. PEBV expression cassettes of RNA1 and RNA2-based VIGS vectors are *pCAMBIA1300* derived plasmids with a CaMV 35S promoter (35 S) and a NOS terminator (T) for transcriptional control, which are inserted between right and left borders (RB and LB) of the T-DNA. The cDNA fragments of *PsCHLI1* and *PsCHLI2* were indicated above. CP, the coat protein coding region.

##
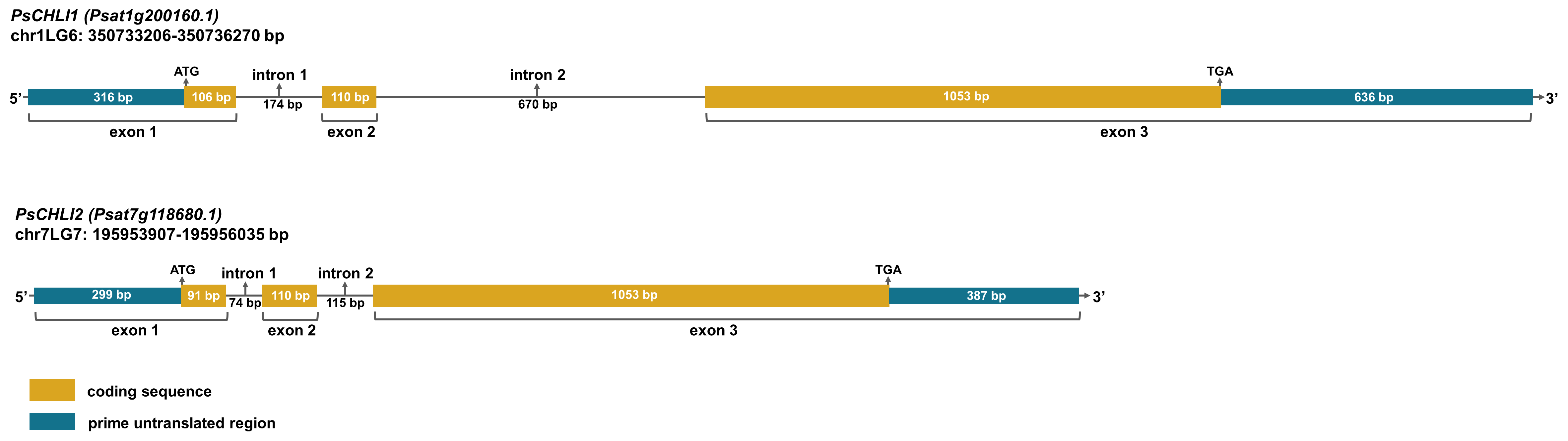
Supplementary Figure 2. Genomic structures of *PsCHLI1* and *PsCHLI2*. *PsCHLI1* and *PsCHLI2* genes were found to be located on chromosomes 1 (Psat1g200160.1, chr1LG6: 350733206-350736270) and 7 (Psat7g118680.1, chr7LG7: 195953907-195956035), respectively (Fig. S1). Both *PsCHLI* genes contained three exons and two introns and same lengths of exon 2 and the coding sequence in exon 3. *PsCHLI1* had longer introns and five-prime (5’) and three-prime (3’) untranslated regions than *PsCHLI2*.


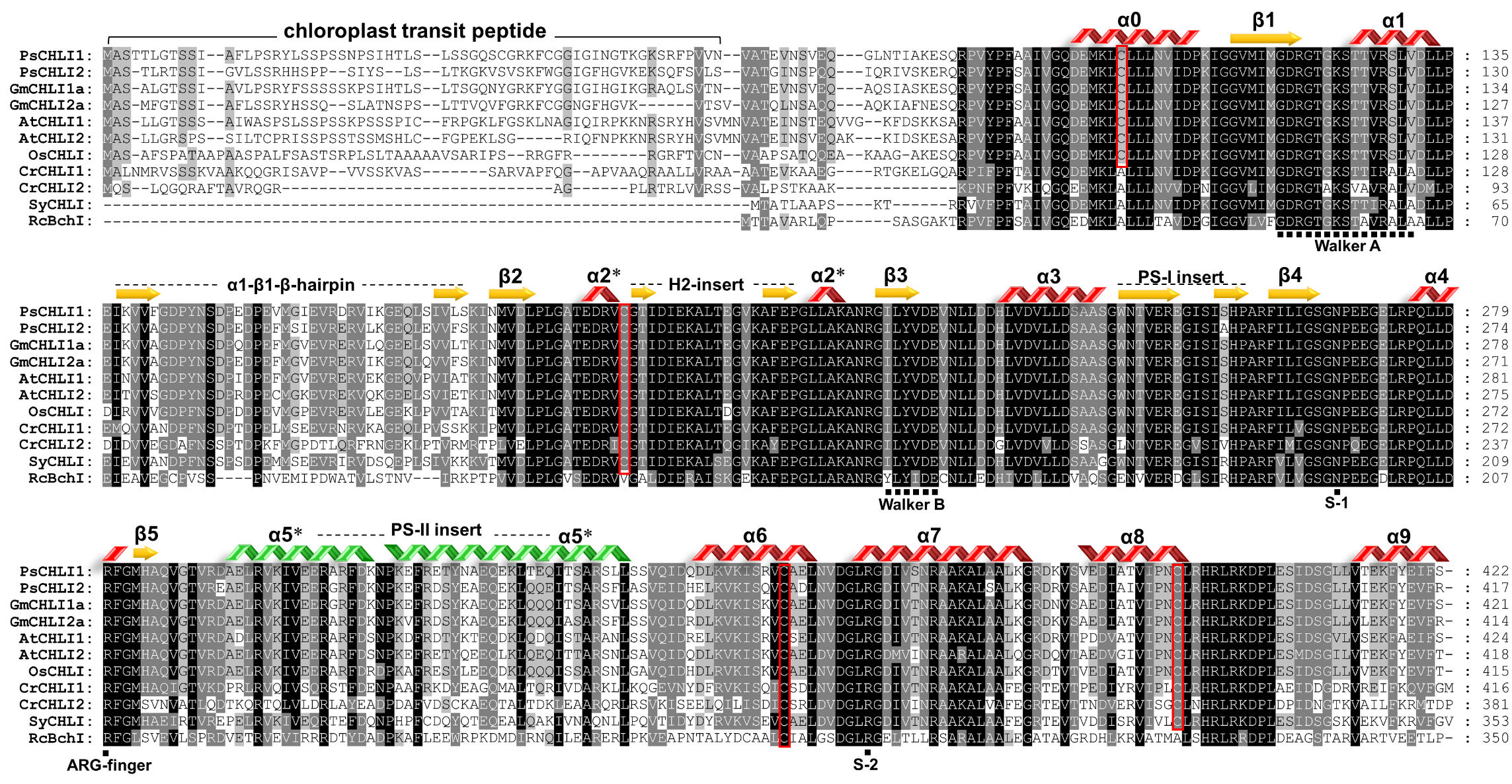
**Supplementary Figure 3.** Structure-based alignment of the amino acid sequences of CHLI/BchI. Sequences were taken from *P. sativum* [PsCHLI1 (AET86637.1) and PsCHLI2 (QHS64157.1)], *G. max* [GmCHLI1a (XP_003543008.1) and GmCHLI2a (XP_003528479.1)], *A. thaliana* [AtCHLI1 (NP_193583.1) and AtCHLI2 (NP_199405.2)], *O. sativa* [OsCHLI (XP_015627981.1)], *C.* *reinhardtii* [CrCHLI1 (XP_001691232.1 and CrCHLI2 (XP_001690873.1)], *Synechocystis sp. PCC6803* [SyCHLI (BAA17166.1)] and *R. capsulatus* [RcBchI (CAA77538)]. Secondary structure elements are shown on top of the alignment (arrows: β-sheets; spirals: α-helices). The positions of characteristic AAA+ motifs are also marked along the sequence. Conserved cysteines are indicated by red boxes.

## Supplementary Figure 4. The amplification efficiencies of the primers. The cDNA template was defined as starting quantity of 32 (2^6^) and diluted by a factor of 2, 4, 8, 16 and 32, and then analyzed by quantitative real-time PCR with the primers for *PsCHLI1*, *PsCHLI2* and *EF-1α* described in Table S2. Regression of Cq values against the log_2_(starting quantity) was calculated. The amplification efficiencies of the primers for *PsCHLI1* (a), *PsCHLI2* (b) and *EF-1α* (c) were calculated by the formula as following: E=2^-1/slope^-1.
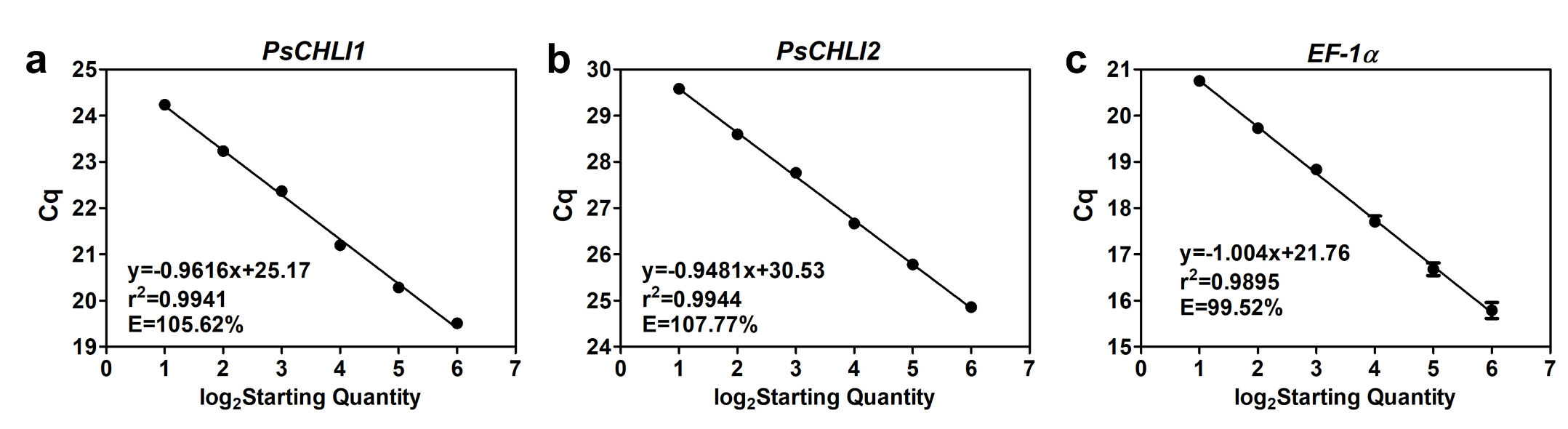


## Supplementary Figure 5. Phenotypes of virus-induced gene silencing (VIGS) plants. *PsCHLI1* and *PsCHLI2* were silenced in pea by a VIGS method. a, VIGS-GFP plant represent a negative control for the effect of virus infection. b, VIGS-PsCHLI plant described in our previous study^16^ was used as positive control, showing the phenotype of yellow leaves. c, VIGS-PsCHLI1 plants showed yellow leaves. d, VIGS-PsCHLI2 plant resembled VIGS-GFP control plants. All plants indicated three independent infiltrations and were observed 3 weeks after infiltration. Error bars indicate the standard error of the mean (SEM) from at least 6 VIGS plants in three independent infiltrations.
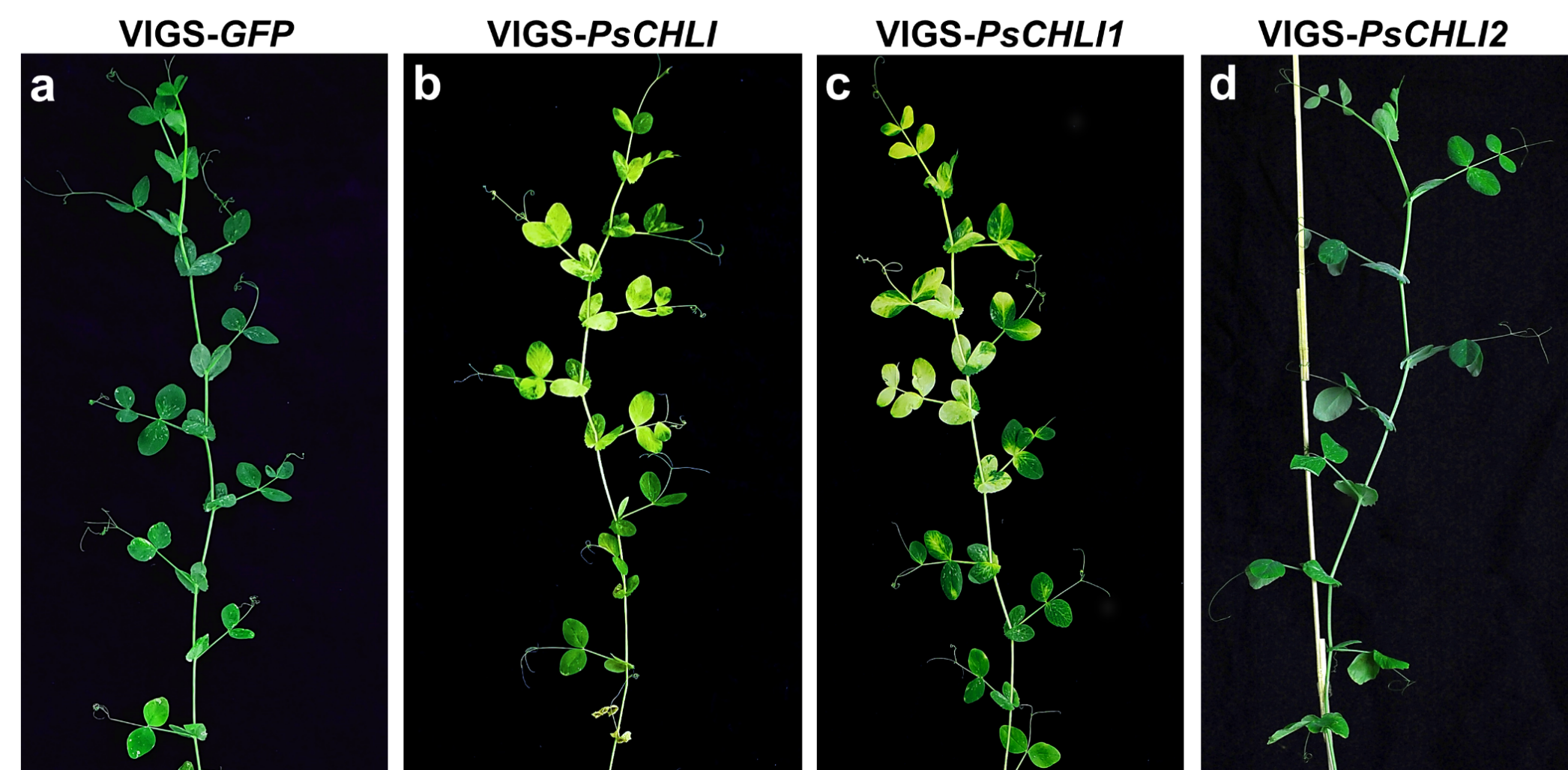


##
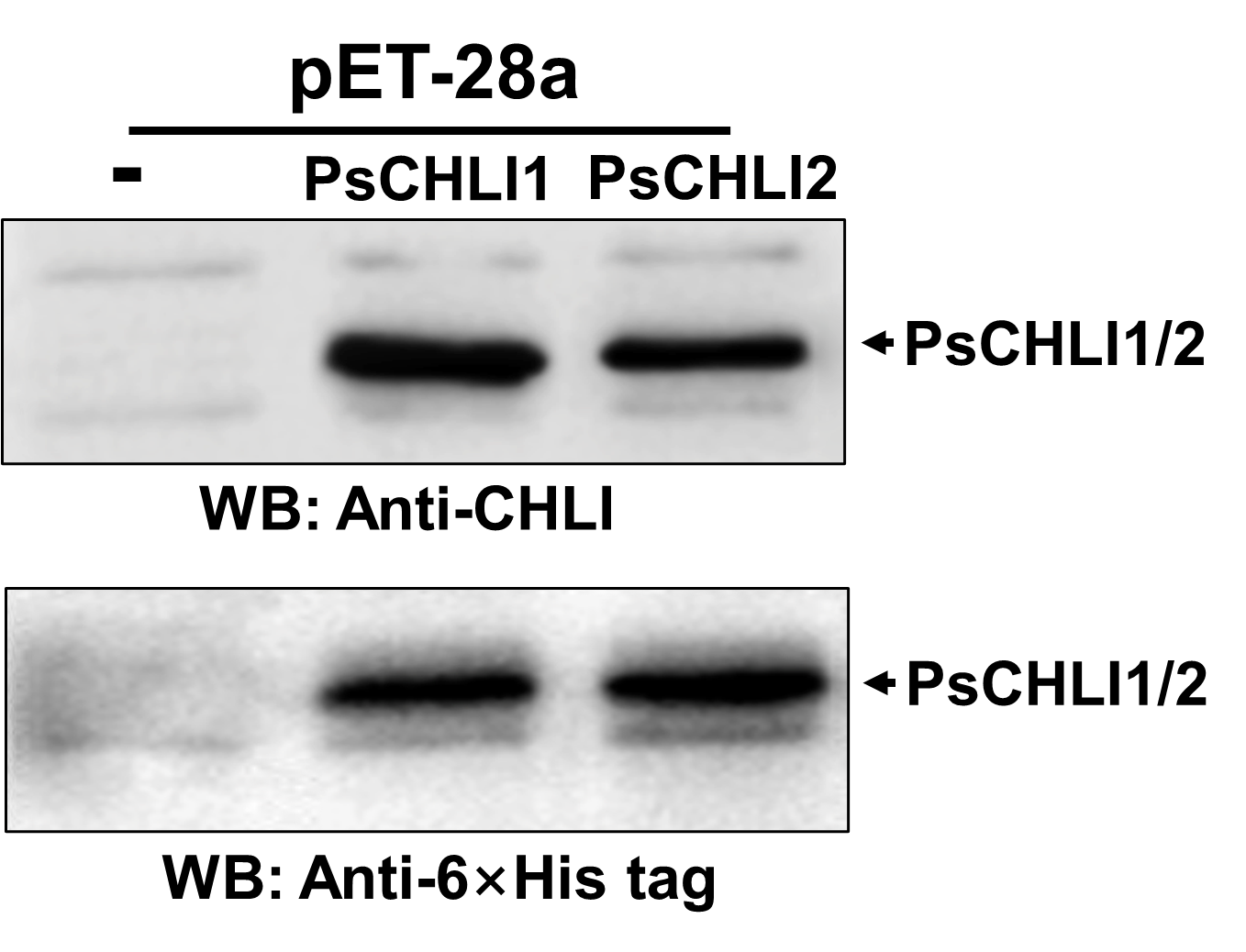
Supplementary Figure 6. Anti-Arabidopsis CHLI1 antibody recognized both recombinant PsCHLI1 and PsCHLI2 overexpressed in *Escherichia coli*. The plasmids of pET28a-PsCHLI1 and pET28a-PsCHLI2 were overexpressed in *E. coli* and the expression products were examined by western blot using a previously verified anti-Arabidopsis CHLI1 antibody^16^ and an anti-His tag antibody (Abcam PLC, Cambridge, MA, USA).

##
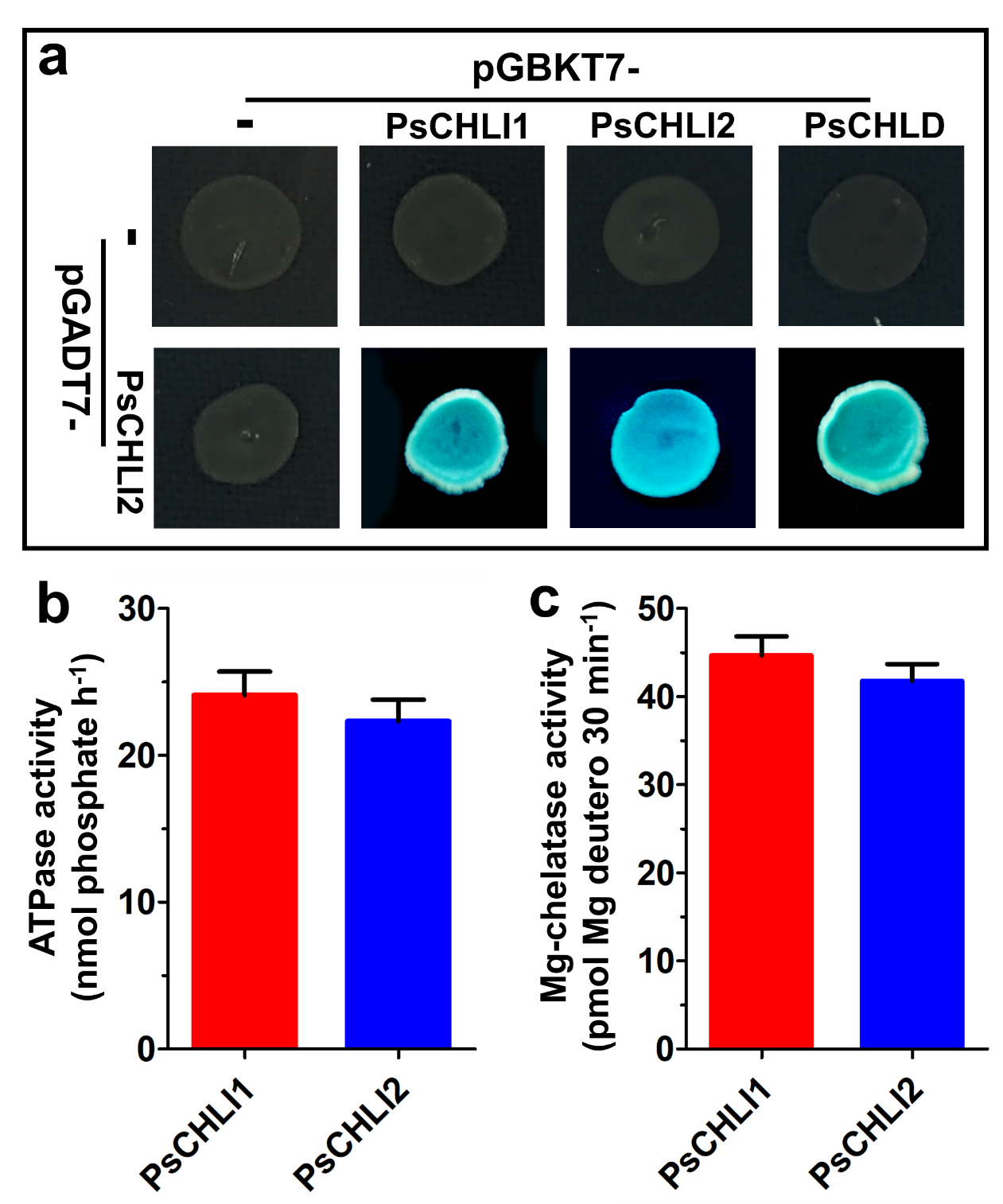
 Suppl ementary Figure 7. Roles of PsCHLI2 in protein-protein interaction and enzyme activities. a, The protein-protein interactions among the PsCHLI1, PsCHLI2 and PsCHLD were determined by the yeast two-hybrid assay. b, 1 μM PsCHLI1 and PsCHLI2 purified from *E. coli* were used for ATPase activity by the Malachite Green colorimetric Assay. c, 0.2 μM PsCHLI1 and PsCHLI2 purified from *E. coli* were combined with recombinant rice CHLD (0.05 μM), CHLH (0.5 μM), and GUN4 (0.5 μM) proteins to reconstitute the Mg-chelatase activity *in vitro* by a stopped fluorometric assay. Data represent the mean ± S.E.M of 9 replications from three independent assays.

##
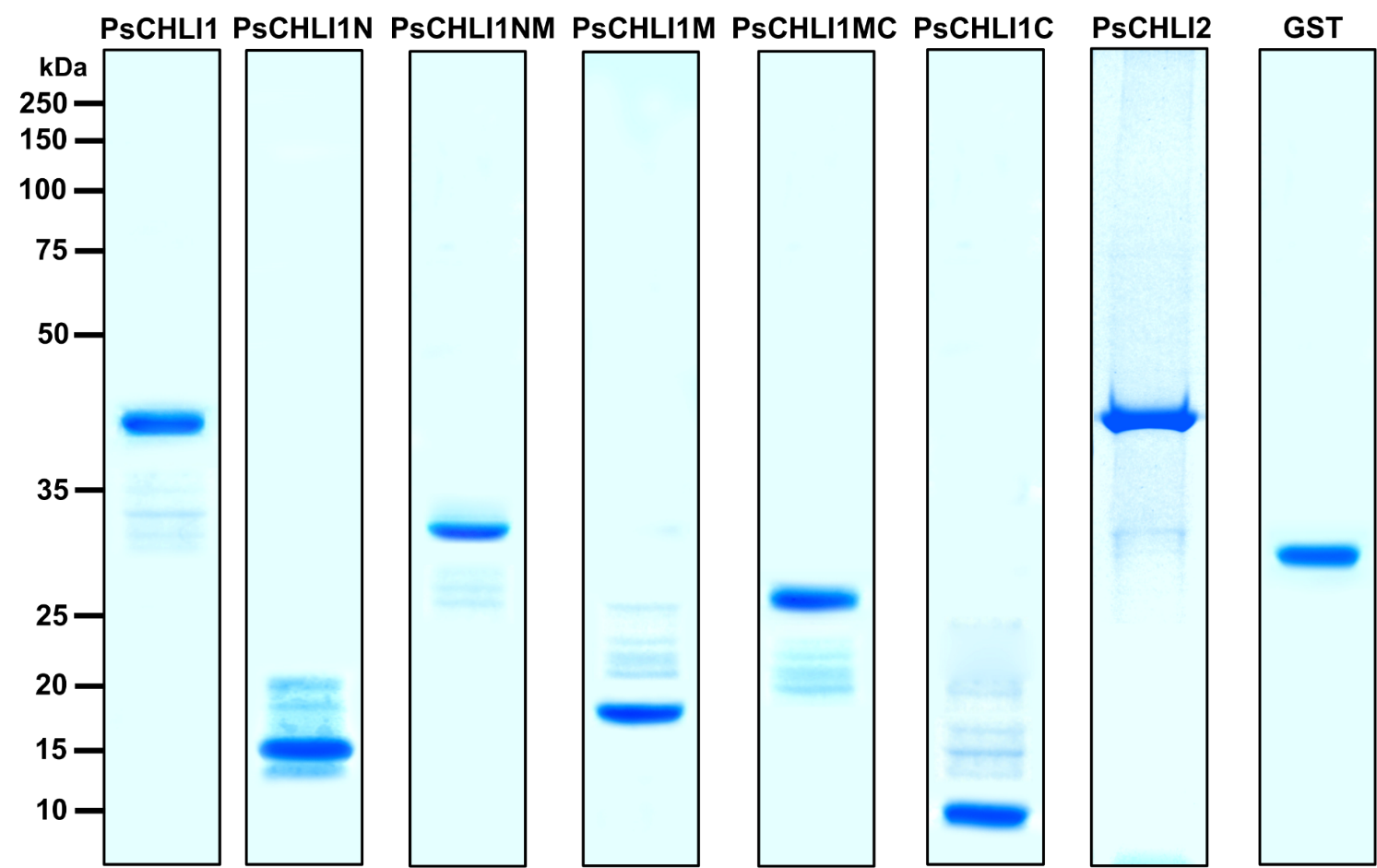
 Supplementary Figure 8. SDS-polyacrylamide gel electrophoresis of the purified recombinant PsCHLI1 fragments, PsCHLI2 and GST protein. The positions of molecular mass markers are indicated at the left.
